# Global, regional, and national burden and trends of schizophrenia among adolescents and young adults aged 15–39 years from 1990 to 2021: an analysis of the global burden of disease study 2021

**DOI:** 10.3389/fpsyt.2025.1682955

**Published:** 2025-11-18

**Authors:** Bo Peng, Feihu Hu, Qin He, Yuluo Tu, Gui Xie

**Affiliations:** 1 Department of Neurology, Nanchang Hongdu Hospital of Traditional Chinese Medicine, Nanchang, China; 2 Department of Psychosomatic Medicine, the Second Affiliated Hospital of Nanchang University, Nanchang, China

**Keywords:** schizophrenia, global burden of disease, incidence, mortality, disability-adjusted life years, socio-demographic index

## Abstract

**Background:**

Schizophrenia imposes a substantial disability burden among individuals aged 15–39 years, yet systematic global studies are lacking. Based on data from the Global Burden of Disease (GBD) 2021 data, this study analyzed trends in schizophrenia among people aged 15–39 years at the global, regional, and national levels from 1990 to 2021.

**Methods:**

Using GBD 2021 data, we analyzed the incidence, prevalence, and disability-adjusted life years (DALYs) of schizophrenia across 204 countries and territories. Age-standardized incidence (ASIR), prevalence (ASPR), and DALY rates (ASDR) with estimated annual percentage changes (EAPC) were calculated and stratified by age, sex, and Socio-demographic Index (SDI). Decomposition analysis assessed driving factors, and a Bayesian age–period–cohort (BAPC) model was used to project trends through 2050.

**Results:**

In 2021, the global numbers of incident cases, prevalence, and DALYs of schizophrenia among people aged 15–39 increased significantly since 1990. ASIR slightly declined, while ASPR and ASDR continued rising. Regionally, South Asia had the highest absolute numbers of new cases, prevalence, and DALYs; Oceania the lowest. Australasia recorded the highest ASIR, ASPR, and ASDR; Eastern Europe had the lowest ASIR; Central Sub-Saharan Africa had the lowest ASPR and ASDR. Nationally, New Zealand had the highest ASIR, Suriname the lowest; Australia had the highest ASPR and ASDR, Somalia the lowest. ASIR showed a nonlinear relationship with SDI, rising with SDI in low (<0.46) and high (>0.61) ranges but decreasing in middle-low (0.46–0.61) range. ASPR and ASDR positively correlated with SDI. Burden was higher in males than females; 20–24 age group had the highest ASIR, 35–39 group the highest ASPR and ASDR. Decomposition showed age-structure changes mainly drove burden decreases; population growth drove increases. BAPC model predicts stable standardized rates but persistently high absolute burden globally by 2050.

**Conclusion:**

From 1990 to 2021, the global impact of schizophrenia on adolescents and young adults increased significantly, with clear variations across regions, countries, age groups, and genders by socio-demographic levels. Thus, countries should tailor mental health policies to their development and resources, prioritizing early screening, intervention, and long-term management in youth to effectively reduce the public health burden of schizophrenia.

## Introduction

Schizophrenia is a chronic, disabling psychiatric disorder characterized by disorganized thinking, perceptual disturbances, emotional dysregulation, and behavioral impairments (1, 2). Although it can affect individuals across all age groups, its incidence is particularly prominent among adolescents and young adults aged 15–39 years (3). This life stage represents a critical developmental period during which cognitive functions mature, educational and career paths are established, and social networks expand (4). The onset of schizophrenia during this phase often results in academic disruption, occupational limitations, and social dysfunction, ultimately exerting profound impacts on individuals, families, and society at large. Therefore, understanding the epidemiological characteristics of schizophrenia within this age group is of great importance for promoting early identification, targeted interventions, and systematic disease management. Establishing effective prevention systems and support mechanisms holds promise for reducing the long-term disease burden and improving the quality of life and social integration of young individuals.

Previous studies on the burden of schizophrenia have primarily focused on all-age populations or specific regions, with a notable lack of comprehensive assessments targeting the global burden, regional heterogeneity, and sociodemographic disparities among individuals aged 15–39 years. Furthermore, earlier research is often limited by outdated data and inconsistent methodologies, rendering it difficult to accurately capture recent trend shifts. For instance, many studies rely on pre-2019 Global Burden of Disease (GBD) data and thus fail to account for the far-reaching effects of emerging factors such as the COVID-19 pandemic and the widespread adoption of digital lifestyles on mental health service systems (5–7). In addition, variations in diagnostic criteria, disparities in healthcare accessibility, and cultural biases may contribute to reporting discrepancies across regions. Stratified analyses based on the Socio-demographic Index (SDI) remain scarce, further limiting the development of evidence-based, targeted intervention strategies.

This study utilized the most recent data from the Global Burden of Disease Study 2021 to systematically assess the epidemiological characteristics and evolving burden of schizophrenia among adolescents and young adults aged 15–39 years from 1990 to 2021. By analyzing incidence, prevalence, disability-adjusted life years (DALYs), and the estimated annual percentage changes (EAPC) of their age-standardized rates, this study aimed to reveal spatiotemporal distribution patterns, population heterogeneity, and associations with SDI. Furthermore, decomposition analysis was employed to quantify the relative contributions of population aging, growth, and epidemiological transition to changes in disease burden, and a Bayesian age-period-cohort (BAPC) model was used to project trends over the next 29 Years. The findings are expected to inform the optimization of global mental health resource allocation and support the development of precision public health strategies tailored to age and region, thereby contributing critical insights to help mitigate the global mental health crisis.

## Methods

### Data source

This study was based on data from the GBD 2021, a comprehensive database that integrates epidemiological information on 371 diseases and injuries and 88 risk factors across 204 countries and territories from 1990 to 2021. The database enables a dynamic and systematic assessment of global and regional health burden trends and their underlying causes. We extracted schizophrenia-related data specific to adolescents and young adults aged 15–39 years from the GBD database, including incidence, prevalence, DALYs, and age-standardized rates (ASR) for each indicator, along with corresponding 95% uncertainty intervals (UI) to ensure statistical robustness and reliability. All primary data were obtained from the official website of the Institute for Health Metrics and Evaluation (IHME), University of Washington (http://ghdx.healthdata.org/) (8). The IHME applies standardized disease classification systems, modeling methods, and adjustment procedures to integrate and harmonize multi-source data from different countries and regions, ensuring consistency and comparability across time and space. As part of the GBD 2021 framework, the data used in this study provide a robust foundation for understanding the epidemiological characteristics and regional heterogeneity of schizophrenia among individuals aged 15–39 years.

### Disease definition

In the GBD 2021 disease classification system, schizophrenia is categorized as a level 3 disease under the level 2 category of "mental disorders," which in turn falls under the broader level 1 category of "non-communicable diseases." As a severe and chronic psychiatric disorder, schizophrenia is primarily characterized by disorganized thinking and reasoning, perceptual disturbances such as hallucinations and delusions, emotional dysregulation, and marked impairments in social functioning and behavioral control. The disease is known for its high disability and relapse rates. In terms of diagnostic coding, the GBD study adheres to the World Health Organization's International Classification of Diseases (ICD) to ensure global consistency and diagnostic accuracy. In the ICD-9 system, schizophrenia corresponds to codes 295.0–295.9, while in the widely adopted ICD-10 system, it falls under the F20.0–F20.9 coding range, covering various subtypes of chronic non-organic psychoses (9). This standardized classification provides a unified diagnostic and data processing framework for measuring the global burden of schizophrenia.

### Decomposition analysis

This study employed decomposition analysis to quantitatively assess the relative contributions of three key components—population aging, population growth, and epidemiological changes—to the changes in schizophrenia burden among adolescents and young adults aged 15–39 years globally from 1990 to 2021. This method overcomes the limitations of conventional trend analyses and allows for the identification of dominant driving factors across different regions, sexes, and age groups (10). The results provide an evidence-based foundation for designing more targeted and differentiated public health interventions. Ultimately, this approach supports decision-makers in optimizing resource allocation and prioritizing the most impactful drivers of burden to more effectively reduce the global health impact of schizophrenia.

### Predictive analysis

This study utilized the BAPC model in conjunction with the Integrated Nested Laplace Approximation (INLA) algorithm to project future trends in the schizophrenia burden among adolescents and young adults aged 15–39 years from 1990 to 2021. The BAPC model, built upon a Bayesian statistical framework, allows simultaneous estimation of age, period, and cohort effects, capturing both their independent and interactive influences on disease trends. This approach provides a more comprehensive understanding of temporal dynamics and generational differences in disease burden. Compared with traditional Markov Chain Monte Carlo (MCMC) methods, the BAPC-INLA framework approximates marginal posterior distributions more efficiently, addressing the computational complexity and convergence issues associated with MCMC in high-dimensional settings. This approach significantly improves model accuracy and stability in both fitting and forecasting. Data preprocessing, model implementation, and results visualization were performed entirely within the R programming environment, ensuring the reproducibility and transparency of the analytic workflow. As a robust predictive tool, the BAPC model demonstrates strong applicability and generalizability in mental health and chronic disease epidemiology (11, 12).

### Statistical analyses

Based on data from the GBD 2021, this study systematically assessed the burden of schizophrenia among adolescents and young adults aged 15–39 years from 1990 to 2021. Standardized epidemiological indicators were used, including age-standardized incidence rate (ASIR), age-standardized prevalence rate (ASPR), and age-standardized disability-adjusted life year rate (ASDR), all reported per 100,000 population with corresponding 95% UI to reflect estimation precision. For temporal trend analysis, a log-linear regression model (ln (ASR) = α + βX + ϵ) was constructed to calculate the EAPC and its 95% confidence interval (CI), with trends interpreted as follows: a rising trend was defined if both the EAPC and its lower CI bound were > 0; a declining trend if the upper CI bound was < 0; otherwise, the trend was considered stable (13). A multidimensional analytical strategy was adopted: (1) Gaussian process regression was used to explore the nonlinear relationship between disease burden and the SDI (14); (2) Spearman rank correlation analysis was conducted to assess the associations between EAPC and SDI, as well as baseline disease burden metrics; (3) Decomposition analysis was performed to quantify the relative contributions of population aging, population growth, and epidemiological changes to variations in incidence, prevalence, and DALYs. In addition, to forecast future burden trends of schizophrenia, the BAPC model combined with the INLA algorithm was applied. This modeling used historical epidemiological data and population projections to simulate the dynamic burden of schizophrenia from 2022 to 2041. All data processing and statistical analyses were conducted using R software (version 4.4.2) and the JD_GBDR platform (V2.37) developed by Jingding Medical. All statistical inferences were based on two-sided tests, with a significance level of α = 0.05, ensuring the scientific rigor and reliability of the results.

### Ethical considerations

This study utilized publicly available data from the GBD repository and did not involve human subjects directly; therefore, ethics committee approval was not required.

## Results

### Global trends

Globally, in 2021, the number of new schizophrenia cases among adolescents and young adults aged 15–39 years reached 979,071.85 (95% UI: 757,449.35–1,226,495.95), representing a notable increase compared to 744,896.86 cases (95% UI: 579,060.68–923,531.86) in 1990. Despite this rise in absolute numbers, the ASIR showed a slight decline, from 33.99 per 100,000 population (95% UI: 26.42–42.14) in 1990 to 32.91 per 100,000 (95% UI: 25.46–41.23) in 2021, with an EAPC of −0.06 (95% CI: −0.09 to −0.03). During the same period, the total number of prevalent schizophrenia cases increased from 7,593,809.70 (95% UI: 5,865,180.85–9,482,333.04) in 1990 to 10,851,383.31 (95% UI: 8,326,542.35–13,650,416.79) in 2021. The ASPR also rose from 346.46 per 100,000 (95% UI: 267.60–432.63) to 364.77 per 100,000 (95% UI: 279.90–458.86), with an EAPC of 0.13 (95% CI: 0.10 to 0.16). Additionally, the total number of DALYs in this age group reached 7,121,448.26 (95% UI: 5,037,205.43–9,642,327.21) in 2021, a substantial increase from 4,987,861.71 (95% UI: 3,587,299.64–6,777,493.72) in 1990. The ASDR rose from 227.57 per 100,000 (95% UI: 163.67–309.22) in 1990 to 239.39 per 100,000 (95% UI: 169.33–324.13) in 2021, with an EAPC of 0.14 (95% CI: 0.11 to 0.17) (**Table 1**).

**Table 1:** Global incidence, prevalence, DALYs, age-standardized rates, and EAPC of schizophrenia among adolescents and young adults aged 15–39 years from 1990 to 2021.

Measure	1990		2021		EAPC(95% CI)
	Number (95% UI)	ASR, per 100,000 (95% UI)	Number (95% UI)	ASR, per 100,000 (95% UI)	
Both					
Incidence	744896.86(579060.68,923531.86)	33.99(26.42,42.14)	979071.85(757449.35,1226495.95)	32.91(25.46,41.23)	-0.06(-0.09,-0.03)
Prevalence	7593809.70(5865180.85,9482333.04)	346.46(267.60,432.63)	10851383.31(8326542.35,13650416.79)	364.77(279.90,458.86)	0.13(0.10,0.16)
DALYs	4987861.71(3587299.64,6777493.72)	227.57(163.67,309.22)	7121448.26(5037205.43,9642327.21)	239.39(169.33,324.13)	0.14(0.11,0.17)
Male					
Incidence	399577.62(311439.94,495693.64)	36.05(28.10,44.72)	526871.20(408506.74,658731.51)	34.90(27.06,43.63)	-0.06(-0.09,-0.03)
Prevalence	4058087.40(3138052.38,5061433.72)	366.10(283.10,456.62)	5805551.25(4459093.67,7293157.06)	384.55(295.36,483.09)	0.12(0.09,0.15)
DALYs	2695056.77(1926113.44,3661948.65)	243.13(173.76,330.36)	3857647.42(2732094.29,5218639.51)	255.52(180.97,345.67)	0.13(0.11,0.16)
Female					
Incidence	345319.25(268523.86,427838.22)	31.88(24.79,39.49)	452200.65(348854.83,568183.25)	30.86(23.81,38.78)	-0.06(-0.09,-0.03)
Prevalence	3535722.30(2727075.99,4423984.12)	326.37(251.73,408.37)	5045832.06(3868955.07,6358209.97)	344.40(264.07,433.97)	0.14(0.11,0.17)
DALYs	2292804.94(1646939.52,3109572.70)	211.64(152.02,287.04)	3263800.84(2307344.77,4422685.01)	222.77(157.48,301.86)	0.14(0.11,0.17)

ASR, age-standardized rates; DALYs, disability-adjusted life years; UI, uncertainty interval; CI, confidence Interval; EAPC, estimated annual percentage change.

### Global trends by gender

**Figure 1:**
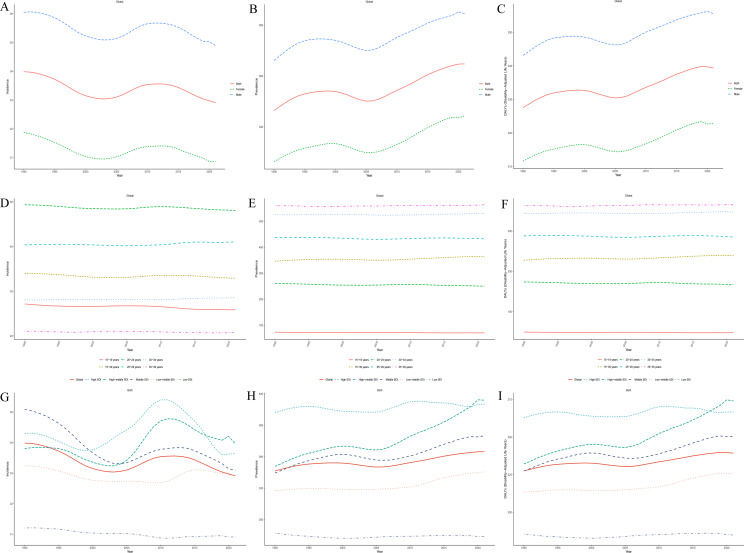
Global trends in the ASIR **(A)**, ASPR **(B)**, and ASDR **(C)** of schizophrenia among adolescents and young adults aged 15–39 years by sex from 1990 to 2021;Global trends in ASIR **(D)**, ASPR **(E)**, and ASDR **(F)** of schizophrenia among individuals aged 15–39 years by age group from 1990 to 2021;Global trends in ASIR **(G)**, ASPR **(H)**, and ASDR **(I)** of schizophrenia among individuals aged 15–39 years across five SDI quintile from 1990 to 2021.

From 1990 to 2021, ASIR of schizophrenia among adolescents and young adults aged 15–39 years showed an overall declining trend globally for both sexes (**Figure 1A**), while the ASPR and ASDR exhibited upward trends (**Figures 1B, C**). Throughout the entire observation period, ASIR, ASPR, and ASDR were consistently higher in males than in females, indicating a greater health loss attributable to schizophrenia among young men. In 2021, the ASIR, ASPR, and ASDR for males aged 15–39 years were 34.90, 384.55, and 255.52 per 100,000 population, respectively, all significantly higher than the corresponding values for females, which were 30.86, 344.40, and 222.77 per 100,000. These figures were approximately 1.13, 1.17, and 1.15 times those of females, respectively. These findings highlight a persistent and marked gender disparity in the burden of schizophrenia, with young males facing a higher risk of disease burden, underscoring the need for gender-sensitive public health strategies.

### Global trends by age groups

Between 1990 and 2021, global ASIR of schizophrenia among adolescents and young adults aged 15–39 years showed an age-dependent pattern. ASIR declined with increasing age in the 15–19 and 20–24 age groups, whereas it increased in the 25–29, 30–34, and 35–39 age groups. For ASPR and ASDR, declining trends were observed in the 15–19, 20–24, and 25–29 age groups, while increasing trends were evident in the 30–34 and 35–39 age groups (**Figures 1D–F**). In 2021, the ASIR was highest in the 20–24 age group, while the ASPR and ASDR peaked in the 35–39 age group. Over the entire study period, the 20–24 age group exhibited the most pronounced changes in burden: ASIR (EAPC = −0.05, 95% CI: −0.07 to −0.03), ASPR (EAPC = −0.09, 95% CI: −0.12 to −0.07), and ASDR (EAPC = −0.08, 95% CI: −0.11 to −0.05), suggesting that schizophrenia burden in this group was the most sensitive to temporal change.

### SDI regional trends

From 1990 to 2021, the ASIR of schizophrenia among adolescents and young adults aged 15–39 years declined across all SDI regions except the high-middle SDI group. The most significant decrease was observed in the middle SDI region, followed by the high SDI region. In contrast, ASPR and ASDR increased in all SDI regions except for the low SDI group, with the most pronounced increases occurring in the high-middle and middle SDI regions (**Figures 1G–I**). By 2021, the high-middle SDI region had the highest ASIR, ASPR, and ASDR among all SDI quintiles, while the low SDI region had the lowest values across all three indicators. This pattern reflects substantial disparities in the disease burden associated with levels of social and economic development (**Supplementary Table 1**).

### Regional trends

**Figure 2:**
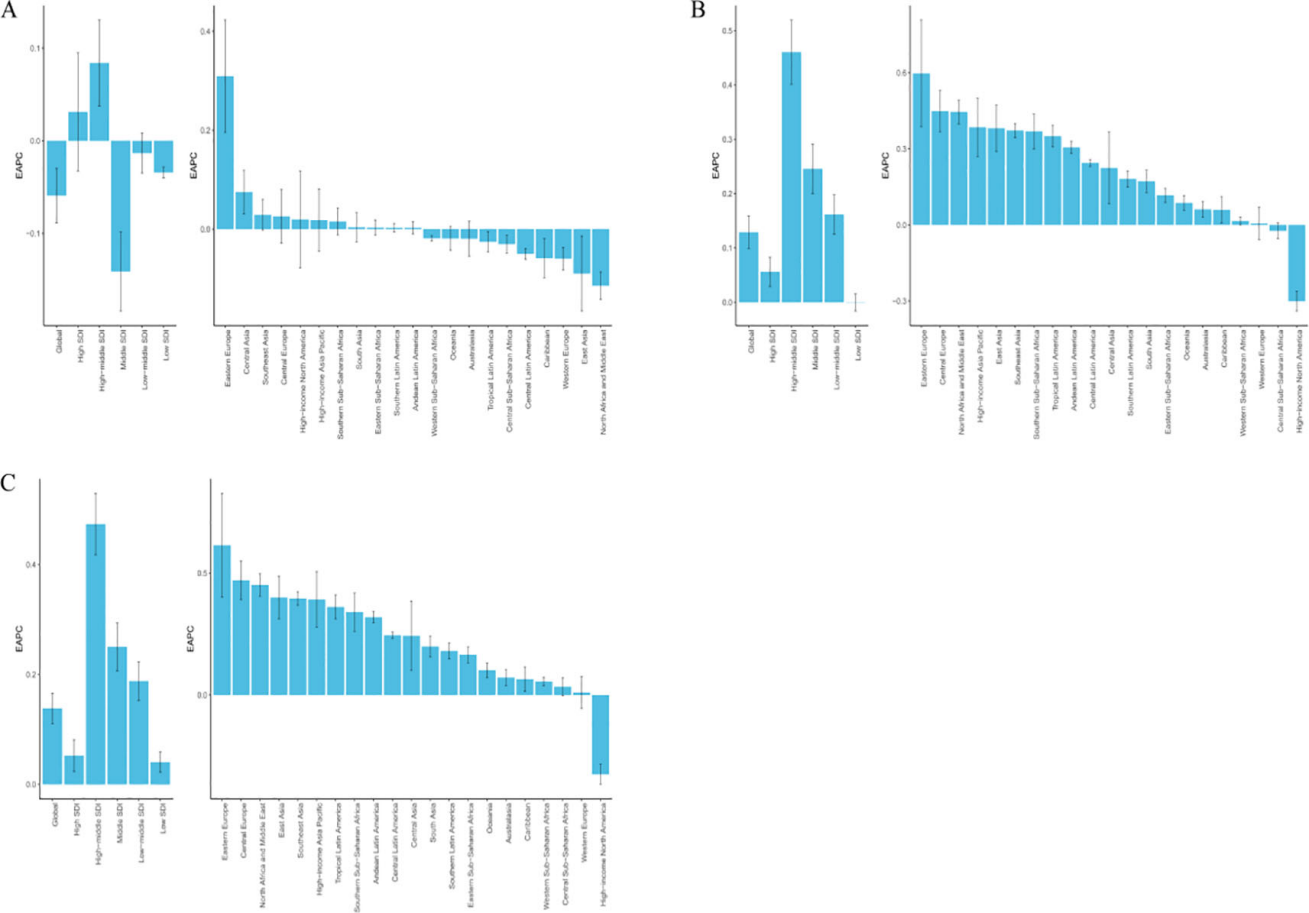
EAPC in age-standardized rates of schizophrenia among adolescents and young adults aged 15–39 years across 21 GBD regions and 5 SDI regions from 1990 to 2021. **(A)** EAPC of ASIR; **(B)** EAPC of ASPR; **(C)** EAPC of ASDR.

**Figure 3:**
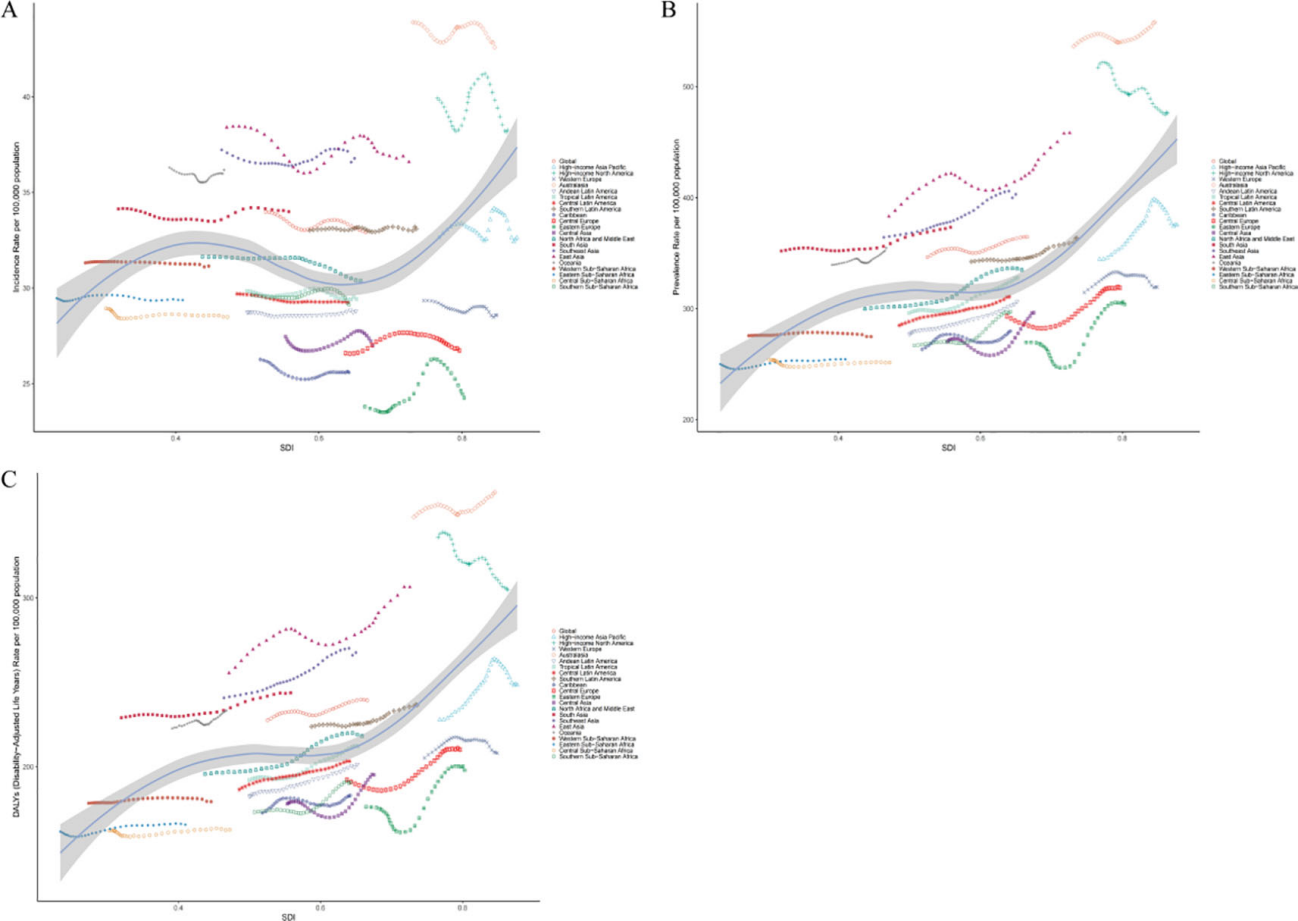
Age-standardized rates of schizophrenia among adolescents and young adults aged 15–39 years across 21 global regions from 1990 to 2021, stratified by SDI. **(A)** ASIR; **(B)** ASPR; **(C)** ASDR.

At the regional level, data from 2021 showed that South Asia was the epicenter of schizophrenia among adolescents and young adults aged 15–39 years, reporting 268,843.82 new cases (95% UI: 206,594.52–337,918.96), 2,952,754.66 prevalent cases (95% UI: 2,256,531.99–3,694,867.85), and 1,928,564.16 DALYs (95% UI: 1,376,382.44–2,614,306.04). In contrast, Oceania reported the lowest burden, with only 2,038.44 incident cases (95% UI: 1,484.91–2,684.60), 19,986.07 prevalent cases (95% UI: 13,897.99–27,093.14), and 13,146.51 DALYs (95% UI: 8,708.28–19,251.23). In terms of age-standardized rates, Australasia had the highest ASIR (42.59 per 100,000, 95% UI: 36.59–49.29), ASPR (558.07 per 100,000, 95% UI: 502.89–625.03), and ASDR (362.59 per 100,000, 95% UI: 276.15–450.31) globally. Conversely, Eastern Europe recorded the lowest ASIR (24.26 per 100,000, 95% UI: 18.66–30.31), while Central Sub-Saharan Africa had the lowest ASPR (251.53 per 100,000, 95% UI: 173.45–347.80) and ASDR (162.67 per 100,000, 95% UI: 107.73–241.62) (**Supplementary Table 1**). Trend analysis from 1990 to 2021 across 21 GBD regions revealed that ASIR increased in 11 regions and decreased in 10. Eastern Europe had the most notable increase (EAPC = 0.31, 95% CI: 0.20–0.42), while the North Africa and Middle East region exhibited the most significant decline (EAPC = −0.11, 95% CI: −0.14 to −0.09). ASPR increased in 19 regions, with Eastern Europe showing the highest increase (EAPC = 0.60, 95% CI: 0.39–0.81); High-income North America was the only region with a decreasing trend (EAPC = −0.30, 95% CI: −0.34 to −0.26). For ASDR, all regions except High-income North America exhibited increasing trends, with the steepest rise observed in Eastern Europe (EAPC = 0.61, 95% CI: 0.40–0.83) (**Figures 2A–C**, and **Supplementary Table 1**). Regarding SDI-related patterns, in 2021, the ASIR of schizophrenia among 15–39-year-olds exhibited a nonlinear association with SDI: it increased with rising SDI in the low (<0.46) and high (>0.61) SDI ranges, but decreased with increasing SDI in the middle-low SDI range (0.46–0.61). Seven regions—including Oceania, Australasia, High-income North America, and Southeast Asia—had ASIR above the global average. In contrast, ASPR and ASDR showed a positive correlation with SDI, consistently increasing with higher SDI levels. Six regions—Australasia, High-income North America, East Asia, Southeast Asia, High-income Asia Pacific, and South Asia—reported ASPR and ASDR above the global mean (**Figures 3A–C**, and **Supplementary Table 1**).

### National trends

**Figure 4:**
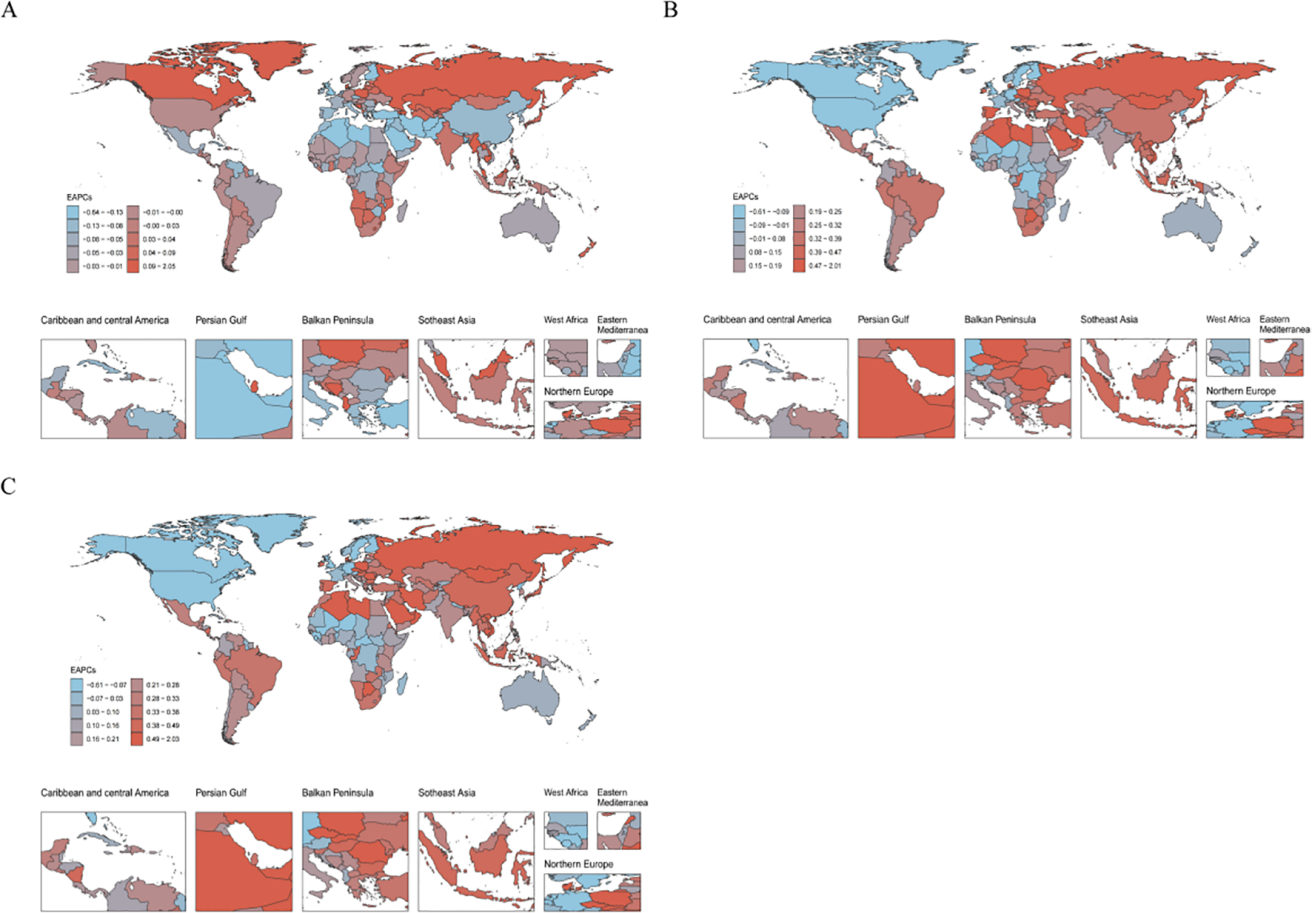
Distribution of the EAPC in age-standardized rates of schizophrenia among adolescents and young adults aged 15–39 years across 204 countries and territories from 1990 to 2021. **(A)** EAPC of ASIR; **(B)** EAPC of ASPR; **(C)** EAPC of ASDR.

At the national level, in 2021, New Zealand reported the highest ASIR of schizophrenia among adolescents and young adults aged 15–39 years, reaching 45.84 per 100,000 population (95% UI: 35.75–56.09), followed by Denmark (43.15 per 100,000, 95% UI: 38.96–45.32) and the Netherlands (41.96 per 100,000, 95% UI: 32.53–52.22). In contrast, the lowest ASIR were observed in Suriname (21.96 per 100,000, 95% UI: 18.27–25.73), the United Kingdom (23.08 per 100,000, 95% UI: 18.07–28.83), and the Republic of Moldova (23.89 per 100,000, 95% UI: 17.53–31.80). For ASPR, Australia (559.43 per 100,000, 95% UI: 507.24–618.48), New Zealand (551.49 per 100,000, 95% UI: 435.19–683.77), and the Maldives (491.96 per 100,000, 95% UI: 348.03–664.19) ranked highest. In contrast, the lowest ASPR were found in Somalia (224.92 per 100,000, 95% UI: 156.48–309.55), the Central African Republic (234.56 per 100,000, 95% UI: 162.80–324.20), and Malawi (235.35 per 100,000, 95% UI: 162.65–323.38). In terms of ASDR, the highest burden was reported in Australia (363.45 per 100,000, 95% UI: 274.67–451.54), New Zealand (358.47 per 100,000, 95% UI: 259.84–470.93), and the Maldives (326.23 per 100,000, 95% UI: 217.67–478.30). The lowest ASDR were observed in Somalia (146.73 per 100,000, 95% UI: 98.13–215.34), the Central African Republic (150.69 per 100,000, 95% UI: 98.82–222.46), and Mozambique (151.80 per 100,000, 95% UI: 97.28–224.44). From 1990 to 2021, trend analysis showed that the United Arab Emirates had the largest decline in ASIR (EAPC = −0.63, 95% CI: −0.88 to −0.39), while Denmark exhibited the most significant increase (EAPC = 2.03, 95% CI: 1.64 to 2.42). For ASPR, the Northern Mariana Islands experienced the greatest decline (EAPC = −0.60, 95% CI: −0.77 to −0.42), whereas Denmark again showed the steepest rise (EAPC = 2.01, 95% CI: 1.68 to 2.33). Similar trends were observed for ASDR, with the Northern Mariana Islands showing the largest decrease (EAPC = −0.60, 95% CI: −0.78 to −0.43) and Denmark the highest increase (EAPC = 1.99, 95% CI: 1.66 to 2.32) (**Figures 4A–C** and **Supplementary Table 2**).

### Decomposition analysis

**Figure 5:**
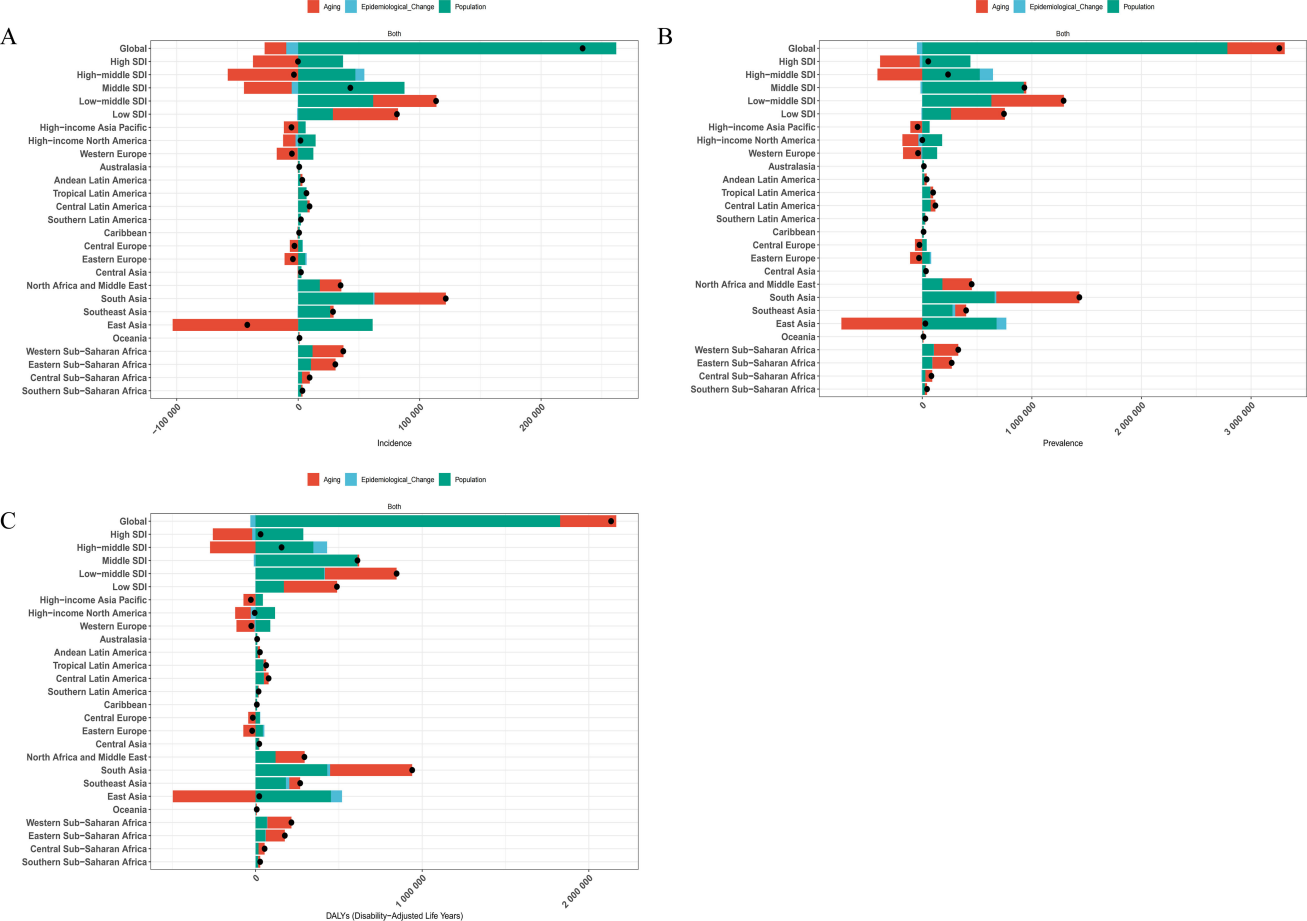
Decomposition analysis of changes in the age-standardized rates of schizophrenia burden among individuals aged 15–39 years from 1990 to 2021, stratified by SDI and 21 GBD regions. **(A)** ASIR; **(B)** ASPR; **(C)** ASDR.

Decomposition analysis revealed the relative contributions of population aging, population growth, and epidemiological changes to trends in schizophrenia incidence, prevalence, and DALYs among adolescents and young adults aged 15–39 years at the global level, across SDI regions, and within the 21 GBD regions. Between 1990 and 2021, a declining trend in the incidence burden of schizophrenia was observed in seven regions—namely, High SDI, High-middle SDI, Central Europe, Eastern Europe, Western Europe, High-income Asia Pacific, and East Asia—where changes in age structure were the predominant negative drivers. This indicates that population aging played a buffering role in reducing new cases of schizophrenia in this age group. In contrast, in regions with rising incidence burdens, population growth was the leading contributor, suggesting that the expanding size of the young population significantly increased the risk of new cases. Regarding prevalence and DALYs burden, Central Europe, Eastern Europe, Western Europe, and High-income Asia Pacific showed notable declines, primarily driven by the suppressive effect of aging populations. Conversely, in regions where prevalence and DALYs burdens increased, population growth remained the dominant driver, reflecting the contribution of an expanding youth base to sustained disease burden. These findings highlight significant heterogeneity in the driving mechanisms of schizophrenia burden trends across regions. In high-income areas, declining burden is largely attributable to demographic shifts brought about by aging, while in low- and middle-income countries and lower-SDI regions, population growth remains a critical upward force. These insights underscore the need for region-specific intervention strategies tailored to the underlying demographic and epidemiological dynamics (**Figures 5A–C**).

### Predicted trends

**Figure 6:**
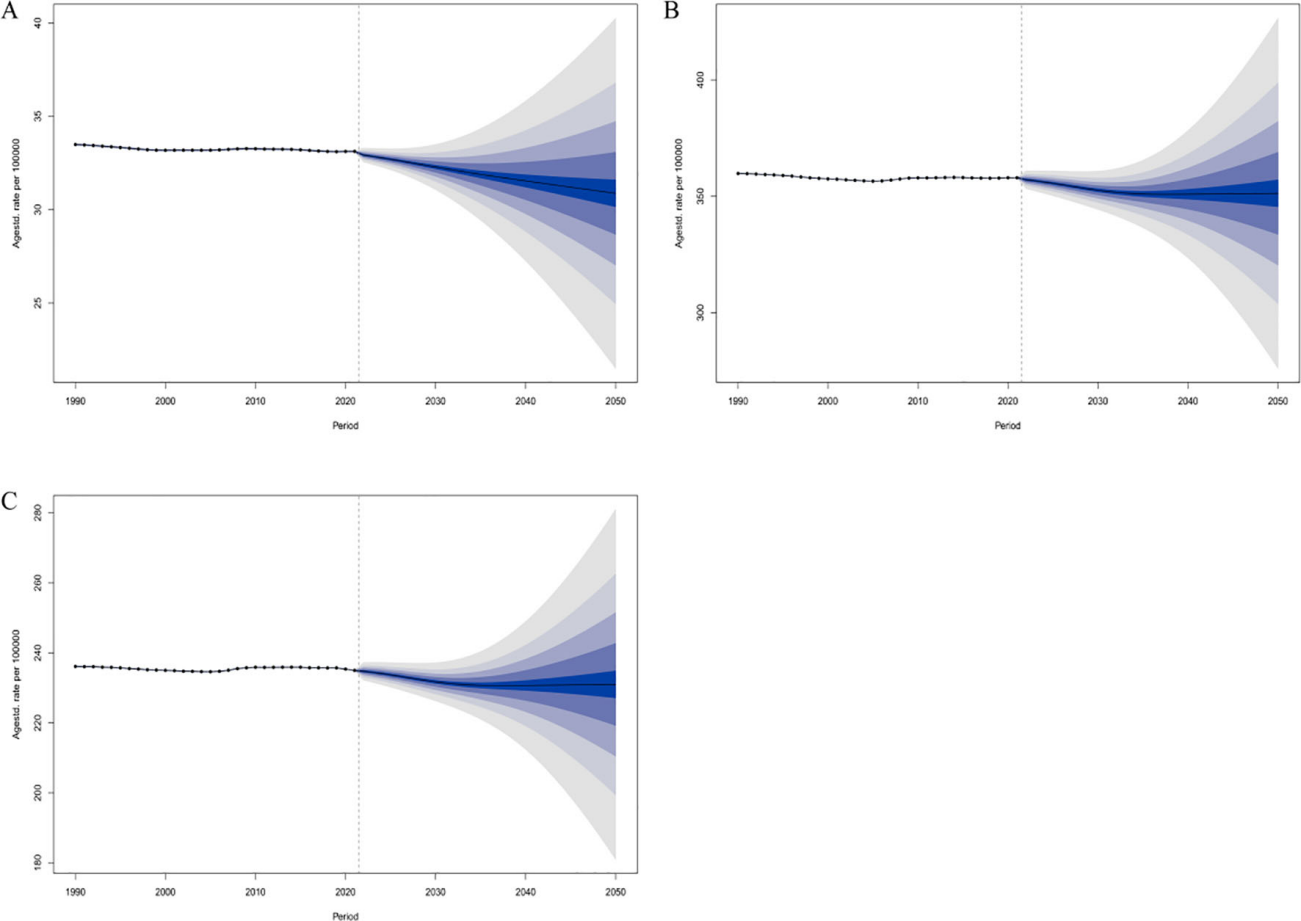
Predicted global changes in age-standardized rates of schizophrenia among individuals aged 15–39 years by 2050. **(A)** ASIR; **(B)** ASPR; **(C)** ASDR.

We used the BAPC model to project the future trends of schizophrenia among individuals aged 15–39 years globally. The results indicated that by 2050, the global ASIR in this age group is expected to decrease slightly to 30.89 per 100,000 population (95% UI: 19.71–42.05), compared with the level in 2021. Over the same period, the ASPR is projected to decline modestly to 351.28 per 100,000 (95% UI: 261.74–440.83). The ASDR is also expected to decrease, reaching 231.00 per 100,000 (95% UI: 171.40–290.59) by 2050. These projected trends suggest that while schizophrenia will continue to impose a significant health burden on adolescents and young adults worldwide, the burden in terms of standardized rates may gradually ease in the coming decades. This trend may reflect the potential effectiveness of existing and future intervention strategies in controlling disease onset and progression. However, given the relatively wide uncertainty intervals, it is essential to remain vigilant against potential risks and to strengthen long-term prevention and control planning for high-risk populations(**Figures 6A–C**).

## Discussion

Schizophrenia is a severe psychiatric disorder that typically begins during adolescence or early adulthood. It is characterized by a prolonged disease course, high disability, and long-term impairment of social functioning, with substantial demands on public health resources (1, 15). Therefore, comprehensively evaluating the evolving epidemiological patterns of schizophrenia in high-risk populations is of critical importance for policymakers and clinicians in developing effective prevention and management strategies. Our study revealed that although the absolute number of new cases, prevalent cases, and DALYs due to schizophrenia among individuals aged 15–39 years has continued to increase globally over the past 32 years, the ASIR showed a slight decline, while the ASPR and ASDR exhibited modest increases. This phenomenon—rising absolute burden alongside stabilizing or even declining standardized rates—may superficially suggest a reduction in schizophrenia risk at the population level but in fact reflects the interplay of demographic dynamics, health system development, and the natural history of the disease. On one hand, the global youth population continues to expand, particularly in low- and middle-income countries (16), which contributes to a marked increase in absolute case numbers despite declining ASIR. On the other hand, improvements in diagnostic capacity and gradual enhancements in mental health services have led to extended patient survival, transforming schizophrenia from an acute disorder into a chronic condition, thereby increasing the accumulation of prevalent cases and DALYs. Moreover, schizophrenia typically manifests during adolescence and early adulthood (6)—a critical life stage characterized by rapid development in social functioning. Its long-term disabling nature exerts profound personal and societal impacts. The observed increase in ASDR also suggests that current interventions remain insufficient in improving long-term functional outcomes. Conversely, the decline in ASIR may reflect phased success in early screening, risk identification, and health education, particularly in high-SDI countries, where mental health systems are more developed and may contribute to reduced incidence or delayed onset (17, 18). However, this progress is not equitably reflected across regions. Mental health system development in low-SDI countries remains relatively underdeveloped, facing challenges such as high underdiagnosis rates, delayed healthcare-seeking, and resource scarcity. Therefore, although the global ASIR has slightly decreased, from the perspective of total burden, schizophrenia remains a long-term and substantial public health challenge among adolescents and young adults. Moving forward, it is imperative to build a more inclusive and resilient global mental health system that addresses risk prevention, early identification, long-term management, and social support—particularly in low-income countries with rapidly growing youth populations—to mitigate the rising burden of schizophrenia.

At the age and sex levels, this study further revealed significant heterogeneity in the burden of schizophrenia. Throughout the study period, males aged 15–39 consistently exhibited higher ASIR, ASPR, and ASDR compared to females. In 2021, the values for males were approximately 1.1 to 1.2 times higher than those for females, indicating that males bear a greater burden of health loss associated with schizophrenia. This sex disparity is consistent with previous epidemiological findings and may be attributed to the interaction of multiple factors (5, 7, 19). Males tend to have an earlier average age of onset, are more likely to be exposed to certain environmental risk factors (e.g., use of psychoactive substances during adolescence, social isolation, traumatic experiences), and may differ from females in symptom recognition and healthcare-seeking behavior, leading to delayed diagnosis and prolonged illness duration (20, 21). Age-specific patterns also showed that schizophrenia peaked during late adolescence and early adulthood—particularly in the 20–24 age group—which recorded the highest ASIR in 2021. This is in line with the "neurodevelopmental vulnerability window" theory, which posits that the prefrontal cortex remains underdeveloped during this stage, making individuals more susceptible to genetic and environmental triggers for psychiatric disorders (22, 23). In contrast, the highest ASPR and ASDR were observed in the 35–39 age group, suggesting that once schizophrenia manifests, the disease tends to persist, and functional recovery is slow, leading to a cumulative burden that intensifies in individuals over 30 and extends into critical stages of life and career. Importantly, from 1990 to 2021, trends varied across age groups. The ASIR in the 15–19 and 20–24 age groups showed a declining or stabilizing trend, possibly reflecting improvements in early identification of psychosis, and greater awareness and support from education systems and families for adolescent mental health. However, the increasing ASPR and ASDR in the over-30 population indicate a growing number of chronic patients within the healthcare system and point to persistent limitations in current treatment approaches regarding remission, relapse prevention, and functional rehabilitation (24, 25). Moreover, this divergence may also reflect ongoing issues with delayed diagnosis, where individuals with mild or subclinical symptoms are only recognized and recorded in later adulthood, thus shifting the burden to older age groups. Overall, age and sex play critical roles in shaping the evolution of schizophrenia burden. These patterns reflect not only biological differences in vulnerability but are also deeply influenced by social behaviors, healthcare system responsiveness, and cultural perceptions. Therefore, future intervention strategies should be more gender-sensitive and age-specific—strengthening early screening and prevention among high-risk youth, and enhancing functional rehabilitation in middle-aged individuals—to ultimately establish a life-course-oriented mental health support system.

The findings of this study indicate significant regional disparities in the burden of schizophrenia among individuals aged 15–39 years worldwide, with a complex association with the SDI. In 2021, South Asia reported the highest number of new and prevalent schizophrenia cases, primarily reflecting the influence of its large population base on the total disease burden. In contrast, Oceania had the lowest absolute number of cases. In terms of standardized indicators, some high-SDI regions demonstrated a higher disease burden than the global average, with Australasia being the most prominent, ranking highest in ASIR, ASPR, and ASDR. This may be attributed to greater disease recognition, more advanced diagnostic systems, and better service accessibility in these regions. Additionally, it may reflect sustained exposure to psychosocial stressors, fast-paced lifestyles, and urbanization-related risk factors among their populations. By contrast, Eastern Europe and parts of sub-Saharan Africa reported the lowest standardized rates, which are more likely the result of low diagnostic coverage, underdeveloped health service systems, and social stigma, leading to substantial underdiagnosis and underreporting, rather than truly lower disease risk. These disparities were further substantiated by time-series analysis across SDI quintiles. From 1990 to 2021, ASIR declined across all SDI regions except the high-middle SDI group, with the most significant reduction observed in the middle-SDI group, followed by the high-SDI group. This suggests that countries with moderate development levels may have achieved substantial progress in reducing the incidence of schizophrenia among youth through expanded primary health infrastructure, broader education access, and improved mental health awareness over the past three decades. In contrast, the lack of decline in ASIR in high-middle SDI regions indicates relatively limited success in risk control. Notably, ASPR and ASDR increased across all SDI quintiles except for the low-SDI group, with the most prominent increases observed in high-middle and middle-SDI regions. This trend may reflect extended survival times, increased chronicity, and enhanced case management, leading to the accumulation of affected individuals in the general population and resulting in higher standardized prevalence and disability burdens. Meanwhile, all three standardized indicators remained lowest in low-SDI regions, which could either reflect a genuinely lower burden or, more plausibly, systemic underestimation due to inadequate mental health services, low diagnostic rates, and persistent stigma. Furthermore, we observed a non-linear relationship between SDI and ASIR: ASIR increased with SDI in both low (<0.46) and high (>0.61) SDI ranges, but decreased with increasing SDI within the middle range (0.46–0.61), forming a "U-shaped" curve. This indicates that schizophrenia risk does not evolve linearly with socioeconomic development but is influenced by multiple interacting factors. In low-SDI regions, traditional social structures, community support, and cultural protection may buffer psychological stress, whereas in high-SDI regions, despite better medical conditions, modern society’s fast pace and competitive pressures may exacerbate mental health risks (26–28). Therefore, mental health intervention strategies should be tailored to the developmental stage of each region. In high-SDI countries, efforts should focus on building psychological resilience among youth, strengthening social support networks, and addressing stress management and functional recovery. In middle and high-middle SDI regions, optimizing chronic disease management systems and extending service coverage is necessary. Meanwhile, in low-SDI regions, the priority should be to enhance service accessibility and diagnostic capacity, expand grassroots mental health resources, and mitigate the expansion of “invisible burden” due to systemic underrecognition.

At the national level, this study revealed substantial disparities in the burden of schizophrenia among individuals aged 15–39 years across countries. These differences are not only influenced by economic development levels but are also closely related to demographic structure, distribution of mental health resources, sociocultural perceptions, and the efficiency of public health policy implementation. In 2021, several high-income countries—such as Australia, New Zealand, and the Maldives—reported ASIR, ASPR, and ASDR values well above the global average. This likely reflects higher rates of disease recognition, longer survival of patients, and more standardized chronic disease management practices, resulting in a statistically higher observed burden. In contrast, several low- and middle-income countries, including Suriname, Somalia, and the Central African Republic, exhibited the lowest ASIR and ASPR globally. While this may appear to indicate a lighter burden, it likely reflects systemic underestimation due to low recognition rates, inadequate healthcare access, and weak reporting infrastructures. Additionally, strong stigma surrounding mental illness in certain cultural contexts may lead individuals to conceal their conditions or fail to seek appropriate care, further contributing to data distortion and management blind spots (29, 30). Notably, from 1990 to 2021, some countries experienced a continuous rise in schizophrenia burden—particularly in high-income settings—possibly due to improved recognition and extended survival that contributed to cumulative statistical burden. In contrast, some Middle Eastern and Pacific Island countries showed a downward trend, which may be linked to regional policy initiatives and the progressive development of mental health systems. These variations indicate that the national burden of schizophrenia is shaped by multiple interwoven factors (31). Therefore, mental health interventions should be tailored to the burden characteristics and available resources of each country. In high-resource, high-burden nations, efforts should focus on early identification, mental health education, and optimization of intervention strategies targeting youth populations. In contrast, low- and middle-resource countries urgently need to strengthen basic mental health service infrastructure, raise public awareness of mental disorders, and promote anti-stigma initiatives at the societal level—ultimately supporting coordinated and equitable progress in global schizophrenia burden reduction.

Through decomposition analysis, we further clarified the driving factors behind regional changes in the burden of schizophrenia. The results showed that in regions where the schizophrenia burden declined between 1990 and 2021—such as high-SDI and some high-middle SDI countries, as well as Central and Western Europe—population aging was the primary negative contributor. This indicates that as populations in these regions aged, the proportion of adolescents and young adults decreased, effectively “flattening the curve” of new cases in this age group. In these areas, the inherent risk of schizophrenia may not have significantly decreased, but the reduction in the size of the youth population helped ease the overall burden. Conversely, in most low- and middle-SDI regions where the burden increased, population growth was identified as the dominant driver, suggesting that the expansion of youth populations directly led to more new cases and a greater cumulative burden of disease. In other words, in many developing countries and regions, the rising burden of schizophrenia has been more closely linked to population growth rather than a substantial rise in incidence rates or worsening disease course. Changes in epidemiological rates (such as incidence or prevalence) were not the main drivers in most regions, and only in a few countries with particularly pronounced burden shifts did they play a notable role. These findings underscore the profound impact of population dynamics on disease burden trends and highlight the need to consider demographic transitions when formulating national mental health strategies. In high-income, aging societies, continued attention should be paid to youth mental health services, but the overall burden may decline due to structural demographic effects. In contrast, in low-income regions experiencing rapid population growth, there is an urgent need to anticipate the rise in schizophrenia cases among expanding youth populations and proactively plan corresponding medical and social support systems.

Our BAPC model provides important insights into future trends. The projections suggest that by 2050, the ASIR, ASPR, and ASDR of schizophrenia among individuals aged 15–39 may decline slightly compared to 2021, indicating a potential easing of the disease burden when measured per 100,000 population. This predicted trend may reflect the gradual effectiveness of global mental health interventions and the impact of demographic shifts. If realized, this would imply a reduction in the per capita risk and disability burden of schizophrenia among young people, and a corresponding decline in its overall share of global health loss. However, it is important to emphasize that the absolute number of young people worldwide—especially in low- and middle-income regions—is still increasing. As such, even with a slight decline in incidence rates, the total number of new and prevalent cases may continue to rise, maintaining substantial demand for healthcare and social support services. Furthermore, the wide uncertainty intervals associated with these forecasts call for cautious interpretation. Potential disruptors—such as the delayed mental health impact of the COVID-19 pandemic (32, 33) and broader socioeconomic changes (34, 35)—could cause future trends to deviate from model projections. Therefore, policymakers should not be complacent but instead view the current modestly optimistic trend as an opportunity to intensify interventions. On one hand, mental health services targeting adolescents and young adults must continue to be strengthened, including early identification, psychological support, standardized pharmacological treatment, and community-based rehabilitation, in order to consolidate and further drive down incidence. On the other hand, anticipating continued growth in the absolute case burden, healthcare resource allocation and social protection mechanisms should be proactively enhanced to address emerging challenges. Additionally, reducing modifiable risk factors—such as curbing substance use among youth and strengthening psychological stress management—holds promise for further lowering the future incidence of schizophrenia.

The strengths of this study lie in the use of the most recent data from the GBD 2021 study, encompassing a 32-year time series, which allowed for a comprehensive assessment of trends in schizophrenia among adolescents and young adults. This study employed a multi-indicator, multi-method approach to analyze disease burden (including both absolute values and standardized rates) in order to enhance the robustness and interpretability of the results. Furthermore, the focus on individuals aged 15–39 years—a critical developmental stage—offers a unique perspective on the mental health challenges specific to the transition from late adolescence to early adulthood. However, several limitations of this study must also be acknowledged. First, the diagnostic criteria and reporting rates for schizophrenia vary significantly across countries. In some low-SDI regions, data are sparse and rely heavily on model-based estimations, which may affect the accuracy of burden estimates. The study’s dependence on the GBD modeling framework means that its findings are inherently influenced by the quality and completeness of the primary data, and the burden in some areas may be either underestimated or overestimated. Second, we did not explore region-specific risk factors or health system characteristics—such as the level of mental illness-related stigma in society, accessibility of mental health services, or substance abuse patterns—that could contribute to the burden of schizophrenia. These factors may help explain some of the observed regional differences but were beyond the scope of this study. Third, although the BAPC model provides valuable insights into future burden trends, projections remain inherently uncertain. The model extrapolates based on past trends and does not fully account for unexpected future events or policy changes (e.g., shifts in mental health investment in the post-COVID era), necessitating cautious interpretation of long-term predictions. Lastly, this study focused exclusively on the schizophrenia burden among adolescents and young adults. The burden among children and older adults was not analyzed. Therefore, the conclusions are primarily applicable to the 15–39 age group, and further research is needed to characterize and compare disease burden across other age cohorts.

## Conclusions

This study, based on data from the GBD 2021, systematically assessed the burden, temporal trends, and driving factors of schizophrenia among adolescents and young adults aged 15–39 years at global, regional, and national levels from 1990 to 2021, and projected trends through 2050. Although the global ASIR of schizophrenia in this age group showed a slight decline, both the ASPR and ASDR continued to increase, with notable disparities by sex and region. High-SDI regions exhibited higher burden rates but a downward trend, while low- and middle-SDI regions experienced a continued rise in burden—largely driven by population growth. Decomposition analysis revealed that changes in age structure were the primary contributors to burden reduction in certain areas, whereas population growth was the main driver of increasing burden in most other regions. Projections using the BAPC model suggest that while standardized indicators may stabilize or slightly decline in the coming decades, the overall burden is expected to remain high. This study underscores that adolescents and young adults are a high-risk and high-burden population for schizophrenia, and should be prioritized in global mental health policy. To effectively mitigate the public health impact, it is crucial to develop regionally tailored and differentiated intervention strategies—strengthening early screening and treatment, improving service accessibility, and enhancing societal investment and awareness in youth mental health. Future efforts should also focus on advancing epidemiological research and surveillance of schizophrenia and promoting cross-sectoral collaboration to achieve substantial reductions in disease burden.

## Data Availability

The original contributions presented in the study are included in the article/**Supplementary Material**. Further inquiries can be directed to the corresponding author/s.
